# Genomic Prediction Including SNP-Specific Variance Predictors

**DOI:** 10.1534/g3.119.400381

**Published:** 2019-08-29

**Authors:** Elena Flavia Mouresan, Maria Selle, Lars Rönnegård

**Affiliations:** * Department of Animal Breeding and Genetics, Swedish University of Agricultural Sciences, Sweden, 75007,; † Department of Mathematical Sciences, Norwegian University of Science and Technology, Norway, 7491, and; ‡ School of Technology and Business Studies, Dalarna University, Sweden, 79188

**Keywords:** BLUP, hglm, CodataGS, external information, Genomic Prediction, GenPred, Shared Data Resources

## Abstract

The increasing amount of available biological information on the markers can be used to inform the models applied for genomic selection to improve predictions. The objective of this study was to propose a general model for genomic selection using a link function approach within the hierarchical generalized linear model framework (hglm) that can include external information on the markers. These models can be fitted using the well-established hglm package in R. We also present an R package (CodataGS) to fit these models, which is significantly faster than the hglm package. Simulated data were used to validate the proposed model. We tested categorical, continuous and combination models where the external information on the markers was related to 1) the location of the QTL on the genome with varying degree of uncertainty, 2) the relationship of the markers with the QTL calculated as the LD between them, and 3) a combination of both. The proposed models showed improved accuracies from 3.8% up to 23.2% compared to the SNP-BLUP method in a simulated population derived from a base population with 100 individuals. Moreover, the proposed categorical model was tested on a dairy cattle dataset for two traits (Milk Yield and Fat Percentage). These results also showed improved accuracy compared to SNP-BLUP, especially for the Fat% trait. The performance of the proposed models depended on the genetic architecture of the trait, as traits that deviate from the infinitesimal model benefited more from the external information. Also, the gain in accuracy depended on the degree of uncertainty of the external information provided to the model. The usefulness of these type of models is expected to increase with time as more accurate information on the markers becomes available.

The identification of a large number of Single Nucleotide Polymorphisms (SNPs) along the genome, as a by-product of the sequencing efforts (*e.g.*, Daetwyler *et al.* 2014) and the development of SNP-chip genotyping technology (Gunderson *et al.* 2005) have made genotyping of thousands of markers affordable at low cost. Meuwissen *et al.* (2001) foresaw these breakthroughs in technology and proposed a new method of selection in animal breeding denoted as Genomic Selection (GS). This method has been tested through simulation studies (Meuwissen *et al.* 2001; Muir 2007) and cross validation with real data in different species such as mice (Legarra *et al.* 2008), dairy cattle (Luan *et al.* 2009; VanRaden *et al.* 2009), aquaculture (Sonesson and Meuwissen 2009) and poultry (González-Recio *et al.* 2009). Nowadays, GS has become part of the routine breeding schemes in dairy cattle (Hayes *et al.* 2009) and other species including pigs (Ostersen *et al.* 2011; Hidalgo *et al.* 2015; Tusell *et al.* 2016) and poultry (Wolc *et al.* 2015).

Several statistical models have been proposed for genomic prediction using whole-genome markers. The most popular method provides best linear unbiased predictions (BLUP) of marker effects (Meuwissen *et al.* 2001) by assuming that the marker effects come from a Gaussian distribution with constant variance and every marker can have an effect on the analyzed trait. This method is referred to either as GBLUP or SNP-BLUP depending on the implementation. Biologically, it seems more reasonable to assume that some of the markers are in linkage disequilibrium (LD) with a causative gene or a quantitative trait locus (QTL) and therefore can capture their effect on the studied trait, whereas some markers are not in LD with any gene and should therefore not capture any effect. To achieve this idea, several methods have been developed to incorporate different prior assumptions on the genetic architecture of the trait. For this family of methods, often referred to as the Bayesian Alphabet (Gianola 2013), it is assumed that the genetic effects of the SNPs follow alternative distributions like a t-distribution (Bayes A) (Meuwissen *et al.* 2001), a double exponential distribution (Bayes LASSO) (de los Campos *et al.* 2009; Usai *et al.* 2009) or a mixture of distributions (*i.e.*, Bayes B, Bayes Cπ, Bayes R) (Meuwissen *et al.* 2001; Habier *et al.* 2011; Erbe *et al.* 2012). The prior assumptions of these methods are rather arbitrary and their performance relies heavily on the model assumptions capturing accurately the true genetic architecture of the trait of interest (Daetwyler *et al.* 2010; Hayes *et al.* 2010).

Whole-genome sequencing of individuals has facilitated the detection of genetic variants that can be used for GS. Currently, in *Bos Taurus* cattle ∼28 million genetic variants have been reported (Daetwyler *et al.* 2013). This large number of polymorphic markers comes with a major challenge in terms of computational speed and memory. One way to deal with this problem is to make use of the biological information available on the markers, *e.g.*, to annotate the markers in classes based on genome location or functionality and prioritize those classes that show a higher probability of containing trait associated markers. Koufariotis *et al.* (2014) showed that protein coding regions explain significantly more variation than similar number of randomly chosen markers across many traits in cattle. Moreover, in a study by Schork *et al.* (2013), the upstream and downstream classes showed significant enrichment in trait associated variants suggesting that these classes can potentially have important regulatory functions. In the same line, Yang *et al.* (2011) stated that genic regions contributed more additive genetic variance than non-genic regions for human traits. However, Do *et al.* (2015) found that the contribution to total genomic variance per SNP among the annotated classes was similar for all regions in a feed efficiency study in pigs.

Several authors have also investigated the predictive ability of models based on annotation classes. Using kernel methods, Morota *et al.* (2014) and Abdollahi-Arpanahi *et al.* (2016) showed that a whole-genome approach provided better predictive ability than that obtained from classes of genomic regions considered separately. Likewise, Do *et al.* (2015) using GBLUP and Bayesian methods (Bayes A, B and Cπ) found that classification of SNPs by genomic annotation had little impact on the accuracy of prediction for feed efficiency traits in pigs.

Apart from genome annotation information, other biological information is available on the SNPs. QTL databases are available for most livestock species (Hu *et al.* 2013) and Genome-Wide Association Studies (GWAS) (Bush and Moore 2012) have identified a great number of trait-associated markers. Moreover, metabolic and signaling pathways (Kanehisa *et al.* 2008; Croft *et al.* 2011; Caspi *et al.* 2012) and gene regulatory networks (Lee *et al.* 2002; Shalgi *et al.* 2007; Hecker *et al.* 2009) can also provide valuable insight to the underlying biology of the traits of interest (Snelling *et al.* 2013). A rather new tool that has been developed to incorporate existing knowledge of the genetic architecture of complex traits into a GS model is BLUP|GA, *i.e.*, “BLUP approach given the Genetic Architecture” (Zhang *et al.* 2014). This tool uses publicly available GWAS results and showed improved prediction accuracies compared to traditional GBLUP and Bayes B methods. Also, a similar approach was developed by Kadarmideen (2014) (system genomic BLUP, - sgBLUP-) where SNPs with known biological role were explicitly modeled in addition to conventional random SNP effects in SNP-BLUP or GBLUP methods. Along with the BLUP approaches, several Bayesian methods were also developed. Bayes Bπ (Gao *et al.* 2015) is a modified version of Bayes B (Meuwissen *et al.* 2001) able to utilize locus-specific priors. In their study, the authors obtained locus-specific priors from variance analysis (ANOVA) based on information from each single marker separately and the results showed improved accuracy and decreased bias compared to Bayes B and Bayes Cπ. In a similar way, MacLeod *et al.* (2016) proposed a modification to the BayesR method (Erbe *et al.* 2012) that incorporates prior biological knowledge. This method provides a flexible approach to improve the accuracy of genomic prediction and QTL discovery taking advantage of available biological knowledge. The basic idea of previously developed methods is to group SNPs into those having a biological function and those with an unknown function. Both the BLUP|GA and BayesBπ methods, also include continuous weights for all, or a subset of markers. For the BLUP|GA method, weights computed using trait-specific GWAS results are used to construct the genomic relationship matrix, whereas in BayesBπ the weights are computed from single-SNP ANOVA analyses.

Although a large number of methods have been developed already for GS, a general BLUP method to include explanatory variables for SNP-specific variances that allow both continuous and class variables seems to be missing. Here we propose a general model using a link function approach within the hierarchical generalized linear model framework (Lee *et al.* 2006). The algorithm proposed by Lee and Nelder (1996) is used, where the hierarchical generalized linear model is fitted by iterating between augmented generalized linear models. With this approach, rather complex models can be fitted using a single deterministic fitting algorithm (see Rönnegård *et al.* 2010a, 2010b).

The aim of the paper is to assess the accuracy for such models including predictors for SNP variances, with special emphasis on the effect of the trait’s genetic architecture and LD structure on estimation accuracy. We present a family of models where the SNP variances can be modeled using both, categorical and continuous predictors, or a combination of the two. The computation time of these models is also studied and a new, faster R package (CodataGS) to fit these models is presented.

## Materials and Methods

### Data simulation

Data were simulated to evaluate the models. Four different scenarios for QTL variance distribution were simulated under three different genetic architectures in which the number of QTL per chromosome was 10, 20 or 100. For each combination of scenario and genetic architecture, 100 simulation replicates were produced. This section describes the simulations in detail.

A base population was simulated of 100 individuals that evolved under random mating for 400 non-overlapping generations (generation -399 to 0) maintaining the population size constant. After the 400 historical generations, two more generations were simulated, still under random mating and expanding the population size from 100 to 200 individuals per generation. Generation 1 was used as training set and generation 2 as validation set. The genome comprised of two chromosomes of 1 Morgan each with 8,800 loci, evenly distributed across the genome. In the base population alleles were coded as 0 or 1 with equal probability resulting in intermediate average allele frequencies. In the first generation, 1,000 loci per chromosome were selected randomly among those loci with a Minor allele frequency (MAF) higher than 0.05 to simulate the SNP marker panel. The same loci were used for validation in generation 2.

To simulate phenotypes in generation 1 (training set), $N_{QTL}$ loci were selected randomly excluding loci that were on the edge of the chromosome and those with a MAF lower than 0.05. In order to simulate different scenarios of genetic architecture underlying the trait, the number of QTL ($N_{QTL}$) varied between 10, 20 and 100 per chromosome. Moreover, the QTL effects, $u_{j}$, *j* = 1, …, $N_{QTL}$, were assumed to be normally distributed with mean 0 and varying variance assigned in one of the following ways:

**Scenario 0 (Sc0):** $u_{j}∼N\left(0,\sigma_{j}^{2}\right),$ where $\sigma_{j}^{2}=e^{1}$
**Scenario 1 (Sc1):** $u_{j}∼N\left(0,\sigma_{j}^{2}\right),$ where $\sigma_{j}^{2}=e^{1}$, with probability 0.5, and $\sigma_{j}^{2}=e^{3}$, with probability 0.5
**Scenario 2 (Sc2):** $u_{j}∼N\left(0,\sigma_{j}^{2}\right),$ where $\sigma_{j}^{2}=e^{1}$, if $u_{j}$ belonged to chromosome 1, and $\sigma_{j}^{2}=e^{3}$, if $u_{j}$ belonged to chromosome 2

Here, *e* is the natural number and therefore the variance can take values between $e^{1}=2.7$ and $e^{3}=20.1.$ The difference between the scenarios Sc1 and Sc2 is that in Sc1 heterogeneous QTL effects are allowed on the same chromosome and may be in linkage disequilibrium with each other. On the other hand, in Sc2 the two different types of QTL are located on different chromosomes to ensure low LD between them.

**Scenario 3 (Sc3):** $u_{j}∼N\left(0,\sigma_{j}^{2}\right),$where $\sigma_{j}^{2}=e^{3f\left(s_{j}\right)}$, $s_{j}$ is the position of QTL *j* and *f* is a function of relative distance to the chromosome edge. Consequently, $\sigma_{j}^{2}$ take values between $e^{1}$ and $e^{3}$. This scenario is motivated by the finding that fitness genes tend to be located closer to the center of the chromosomes (see *e.g.*, Carneiro *et al.* (2009) and references therein).

For each scenario, the three separate genetic architectures were simulated, *i.e.*, with 10, 20 or 100 QTL per chromosome. In order for the results from the different scenarios and genetic architectures to be comparable, the total genetic variance was scaled to 1.0. In this way, the obtained traits were either controlled by a small number of QTL with medium-large effects or by a large number of QTL with small effects.

In generation 1 (training set) phenotypes were simulated for all 200 individuals as:

$$y_{i}=\mu +\sum_{j=1}^{N_{QTL}}Z_{QTL,1ij}u_{j}+e_{i},$$

where $y_{i}$ is the phenotype of individual *i*, *μ* is a fixed effect which was set equal to 0, $Z_{QTL,1ij}$ is the genotype for the *j^th^* QTL coded as 0, 1 or 2 for the homozygote, heterozygote and the alternative homozygote respectively for individual *i* in generation 1, $u_{j}$ is the simulated normally distributed *j^th^* QTL effect as described above, and $e_{i}$ is the residual effect of the *i^th^* individual normally distributed with mean 0 and the appropriate variance $\sigma_{e}^{2}$ in order to create a trait with heritability of 0.2.

Generation 2 was used as validation set where true genomic breeding values (TBVs) were computed as:

$$TBVs_{i}=\sum_{j=1}^{N_{QTL}}Z_{QTL,2ij}u_{j},$$

where $Z_{QTL,2ij}$ is the QTL genotype for QTL *j* and individual *i* for this generation.

### Genomic evaluation

To estimate the SNP effects, the marker panel of 1,000 SNPs per chromosome mentioned above was used and the following model was assumed:

$$y_{i}=\mu +\sum_{j=1}^{p}Z_{ij}\upsilon_{j}+\epsilon_{i},$$ (1)

where $y_{i}$ is the phenotype of individual *i*, *μ* is a fixed effect, *p* is the total number of SNPs, $Z_{ij}$ is the genotype of the SNP *j* for individual *i* coded as 0, 1 or 2, $\epsilon_{i}∼N(0,\sigma_{\epsilon }^{2})$ is the residual effect, and

$$\upsilon_{j}∼N(0,\tau_{j}^{2})$$ (2)

is the *j^th^* SNP effect normally distributed with mean 0 and variance

$$\tau_{j}^{2}=e^{\alpha +\beta x_{j}},$$ (3)

where $\alpha +\beta x_{j}$ is a linear predictor for the SNP-specific variance the components of which are explained in the following section.

### Evaluation models

The linear predictor for variance ($\alpha +\beta x_{j}$) allows to incorporate any type of external information about the SNP variance, making it possible to assign the same variance for all SNPs, a subgroup of SNPs or assign a unique variance for each SNP. We used this linear predictor for variance to introduce external information on the SNPs into the models and the predictive performance of different prior assumptions was tested. The log link ensures a positive variance (Aitkin 1987; Lee and Nelder 1998) and due to its computational robustness is a common choice of link function in variance modeling (Jaffrezic *et al.* 2000; Sorensen and Waagepetersen 2003; Rönnegård *et al.* 2010a). By using a Gamma generalized linear model with a log link, the score function for this model is equivalent to the score function of the REML likelihood in a linear mixed model (Lee and Nelder 1996, Lee *et al.* 2017 page 91) and therefore produces REML estimates of the variance components. Furthermore, especially for variances close to zero the likelihood will be more symmetric on a logarithmic scale than on an untransformed scale, and thereby gives better standard errors for the fitted variance components.

The models tested in this study were:

1. **SNP-BLUP**: In the traditional model the variance of the markers is assumed to be equal for all markers and therefore $x_{j}=0$ in the linear predictor for the variance for all markers.
2. Categorical models (**W10**, **W20** and **W40**): For these models the genome was divided into non-overlapping windows of 10, 20 or 40 SNPs. Then, all the SNPs within a given window were given the value $x_{j}=1$ if they contained a QTL and $x_{j}=0$ if they did not. Hence, a study with known regions harboring the QTL was mimicked, where these regions were known with varying degree of uncertainty.
3. Continuous model (**LD**): For this model, following Yang and Tempelman (2012) and Rönnegård and Lee (2010), the linkage disequilibrium (LD) between a SNP and a QTL was calculated as $r^{2}=D^{2}/(p_{S}p_{s}p_{Q}p_{q})$, where $D=f_{SQ}f_{sq}-f_{Sq}f_{sQ}$ (Falconer and Mackay 1996), $p_{S}$, $p_{s}$, $p_{Q}$ and $p_{q}$ are the allele frequencies of the SNP and QTL, $f_{SQ}$, $f_{sq}$ are the homozygous haplotype frequencies and $f_{Sq}$, $f_{sQ}$ are the heterozygous haplotype frequencies. Then, each SNP was assigned the value of $x_{j}=\sum_{k=1}^{N_{QTL}}r_{jk}^{2}$. The relationship between SNPs and QTL was modeled in such way that markers in higher LD with one or more QTL would be given more importance in the model compared to other markers not in LD with any QTL.
4. Combination of categorical and continuous models (**W10-LD**, **W20-LD** and **W40-LD**): In these models the genome was divided into windows as in the previous categorical models but the SNPs located within a window that harbored a QTL were given the value of the LD with the QTL instead of 1. The model could, therefore, differentiate between SNPs not only based on location but also based on the relationship with the real QTL.
  Table 1 gives an overview of all simulated scenarios and models tested. Each scenario was simulated with 10, 20 and 100 QTL per chromosome as described previously.

**Table 1:** SUMMARY OF MODELS TESTED FOR EACH SCENARIO OF GENETIC ARCHITECTURE SIMULATED

*Models**^a^* *Scenario**^b^*	Sc0	Sc1	Sc2	Sc3
***SNP-BLUP***	**+**	**+**	**+**	**+**
***W10***	**+**	**+**	**+**	**+**
***W20***	**+**	**+**	**+**	**+**
***W40***	**+**	**+**	**+**	**+**
***LD***	**+**	**+**	**+**	**+**
***W10-LD***	**+**	**+**	**+**	**+**
***W20-LD***	**+**	**+**	**+**	**+**
***W40-LD***	**+**	**+**	**+**	**+**

a W10= categorical model with window of 10 SNPs, W20= categorical model with window of 20 SNPS, W40= categorical model with window of 40 SNPS, LD= continuous model with LD estimates, W10-LD= combined model with window of 10 SNPs and LD estimates, W20-LD= combined model with window of 20 SNPs and LD estimates, W40-LD= combined model with window of 40 SNPs and LD estimates.

b Sc0= simulation scenario 0, Sc1= simulation scenario 1, Sc2= simulation scenario 2, Sc3= simulation scenario 3.
5. Additional models (**W10-2var**, **W20-2var**, **W40-2var**, **Dis**, **W10-Dis**, **W20-Dis** and **W40-Dis**): The previously described models include external information on the physical location of the QTL relative to the SNPs or/and the relationship of the SNPs with the QTL but they do not include any information about the QTL variance. Therefore, a few additional models were created based on the particular parameters used for the simulation of each genetic architecture scenario. These models are defined as follows.
  - For the scenarios where the QTL effects came from distributions with two different variances (Sc1 and Sc2) we assumed this information was known and we expanded the linear predictor to $\alpha +\beta x_{j1}+\gamma x_{j2}$ in order to accommodate for more variances (in the models W10-2var, W20-2var, and W40-2var). The genome was divided in non-overlapping windows as before and SNPs associated with a QTL with variance $\sigma_{j}^{2}=e^{1}$ was assigned $x_{j1}=1$ and $x_{j2}=0$, while if it was associated with a QTL with variance $\sigma_{j}^{2}=e^{3}$ it was assigned $x_{j1}=0$ and $x_{j2}=1$. If a SNP was located within a window with no QTL then both $x_{j1}$ and $x_{j2}$ had a value of 0.
  - For Sc3, we used the distance of the markers from the edge of the chromosome as external information either as a continuous variable (Dis) or within windows (W10-Dis, W20-Dis and W40-Dis), since the QTL variances were simulated in the same way.

### German Holstein population data

To demonstrate the model on real data, we used a German Holstein genomic prediction population consisting of 5024 bulls (Zhang *et al.* 2015). Three traits were measured, where the first two had highly significant QTL from a GWAS. Including this information as explanatory variables for the SNP-specific variances was expected to improve genomic selection. We were also able to compare our results with Zhang *et al.* (2015), who have developed the algorithm BLUP|GA that includes information about genetic architecture by building trait-specific genomic covariance matrices.

All bulls had been genotyped and we used the 42,373 SNPs with minor allele frequency above 0.01. For the three traits, which were milk yield, milk fat percentage and somatic cell score, Zhang *et al.* (2015) provide highly reliable estimated breeding values (EBVs) for all bulls from previous studies (Hu *et al.* 2013; Zhang *et al.* 2014). The EBVs for milk yield and milk fat percentage were used as phenotypes.

We chose to fit the model 1) **SNP-BLUP** and models 2) **W11** and **W41**, with windows of size 11 and 41 SNPs centered around candidate QTL peaks. To find candidate QTL, we performed GWAS, correcting for genomic relationship using estimated residual and additive genetic variance from GBLUP. All SNPs from GWAS with p-value less than 10¯^5^ were considered a candidate QTL. For milk yield we identified 6 candidate QTL peaks and for the fat percentage we identified 5 candidate QTL peaks, which were used as the center of the windows.

### Hglm method and CodataGS

The estimation of the SNP effects was performed by fitting the model described by equations 1-3 that allows both continuous and categorical predictors for the SNP-specific variances, or a combination of continuous and categorical predictors. We tested a few examples of external information on the SNPs and these models were fitted using the **hglm** package in R (Rönnegård *et al.* 2010b). In the **hglm** package the linear predictor for variance $\alpha +\beta x_{j}$ is specified using the *X.rand.disp* option in the hglm function and the function estimates SNP effects (example of the command line to call the hglm function with the option *X.rand.disp* can be found in the Supplementary File S1 line 156).

When the number of markers largely exceeds the number of individuals, the computational speed and memory requirements can be improved by fitting individual effects (*i.e.*, EGBVs) in an equivalent model instead of SNP effects (Strandén and Garrick 2009; Shen *et al.* 2013). This equivalent model, which uses the external information on each SNP in the same way as in the **hglm** package, was implemented in the R package **CodataGS** and is available on CRAN (https://cran.r-project.org/web/packages/CodataGS). The theory is explained in the Supplementary File S3. The CodataGS R package was used for the analysis of the German Holstein population data.

### Accuracy

The predictive ability of all models was evaluated as the correlation of the estimated genomic breeding values (EGBVs) and the true genomic breeding values (TGBVs) for the validation set (Generation 2). For each simulation setup, 100 replicates were generated. The convergence of the models varied from 71 to 100% and results are presented for those replicates where all models converged. For the German Holstein population, we performed a fivefold cross-validation with all bulls randomly separated in four groups of 1005 and one group of 1004 with both model 1) and 2). Each group served as a test set while the rest of the groups were used to estimate the SNP effects. The predictive ability was measured as the correlation between the EBVs and the phenotypes of the testing individuals.

### Data availability

Simulation of the data that support the findings is possible through the attached simulation code in File S1 and File S2 (Functions for the simulation) deposited at figshare. The simulation code and the methodology described previously are sufficient to reproduce the results of this study. The analysis program CodataGS used to apply the alternative models on the Holstein dataset is available at https://cran.r-project.org/web/packages/CodataGS. Supplemental material available at FigShare: https://doi.org/10.25387/g3.9247832.

## Results

**Figure 1:**
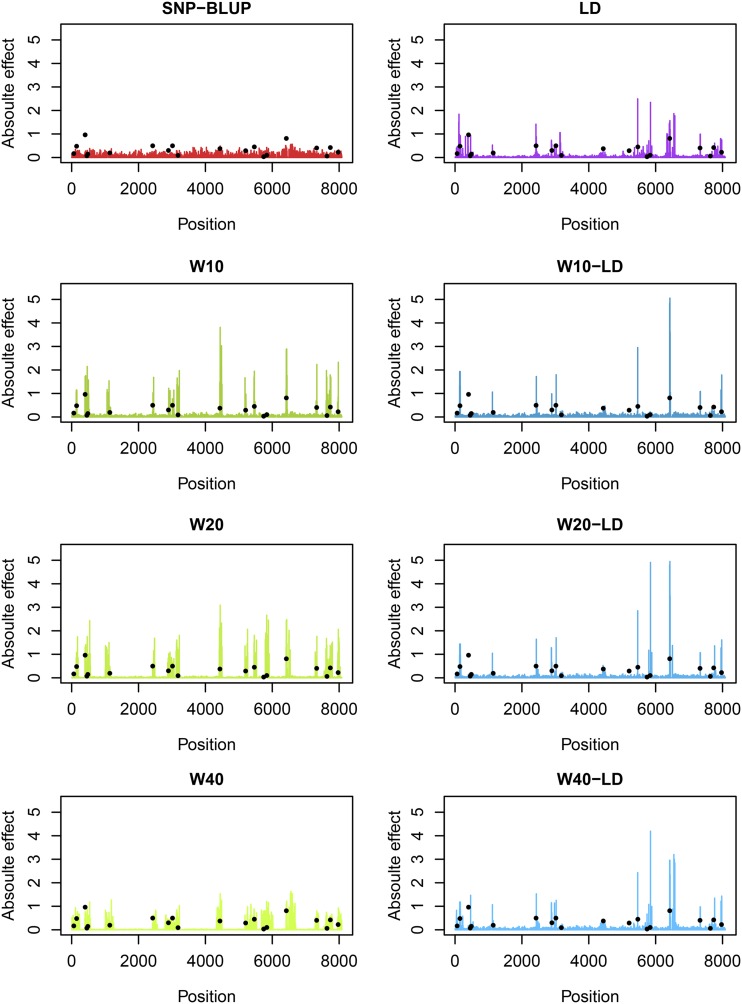
Simulated QTL effects (black dots) and fitted SNP effects under SNP-BLUP and 7 alternative models (Categorical: W10, W20 and W40, Continuous: LD, Combination: W10-LD, W20-LD and W40-LD) for one simulation replicate under simulation scenario Sc0 with 10 QTL per chromosome underlying the trait.

Table 1 contains different versions of the model tested. The fitted SNP effects obtained from **hglm** for one simulation replicate under scenario Sc0 with 10 QTL per chromosome are presented in Figure 1. The R code to reproduce Figure 1 is found in Supplementary File S1 (along with File S2). The results show how the fitted SNP effects may change between model specifications. For example, it can be observed that with increasing window size the estimated effects tend to be spread between more SNPs.

### Model performance

Table 2 shows the accuracies of the predicted EGBVs in the validation set (generation 2) for scenario 0 (Sc0) with 10 QTL per chromosome underlying the trait. In general, the alternative models performed better than SNP-BLUP. The categorical models yielded higher accuracies compared to the SNP-BLUP model by 14.3% (0.670 ± 0.013), 11.9% (0.656 ± 0.012) and 8.4% (0.635 ± 0.012) for the models W10, W20 and W40, respectively. Nonetheless, we observe that the advantage of the categorical models over the SNP-BLUP decreased with increasing window sizes. Moreover, the continuous model (LD) resulted in higher accuracy than the SNP-BLUP or the categorical models with an increase of 22.4% (0.717 ± 0.011) in accuracy with respect to the SNP-BLUP. Similarly, the combination models performed 20.6% (W10-LD, 0.707 ± 0.013) 21.8% (W20-LD, 0.714 ± 0.013) and 23.2% (W40-LD, 0.722 ±0.013) better than the SNP-BLUP model. Contrary to the categorical models, the combination models maintained the gain in accuracy with increasing window size. The alternative models provided unbiased predictions while the SNP-BLUP showed upward bias (Table 2). Finally, the mean squared error of prediction (MSEP) in the validation set improved with the alternative models compared to the SNP-BLUP, indicating that predictions are closer to the true breeding values in the alternative models compared with the SNP-BLUP.

**Table 2:** ACCURACY AND BIAS OF THE PREDICTED EGBVS IN THE VALIDATION SET (GENERATION 2) FOR THE SCENARIO 0 (SC0) WITH 10 QTLS PER CHROMOSOME UNDERLYING THE TRAIT

Models*^a^*	Accuracy (r)	Bias (b)
**SNP-BLUP**	0.586 ^(0.010)^	1.213 ^(0.089)^
**W10**	0.670 ^(0.013)^	1.003 ^(0.044)^
**W20**	0.656 ^(0.012)^	1.014 ^(0.048)^
**W40**	0.635 ^(0.012)^	1.030 ^(0.045)^
**LD**	0.717 ^(0.011)^	1.024 ^(0.041)^
**W10-LD**	0.707 ^(0.013)^	1.050 ^(0.053)^
**W20-LD**	0.714 ^(0.013)^	1.044 ^(0.050)^
**W40-LD**	0.722 ^(0.013)^	1.028 ^(0.042)^

a W10= categorical model with window of 10 SNPs, W20= categorical model with window of 20 SNPS, W40= categorical model with window of 40 SNPS, LD= continuous model with LD estimates, W10-LD= combined model with window of 10 SNPs and LD estimates, W20-LD= combined model with window of 20 SNPs and LD estimates, W40-LD= combined model with window of 40 SNPs and LD estimates.

### Effect of number of simulated QTL

**Figure 2:**
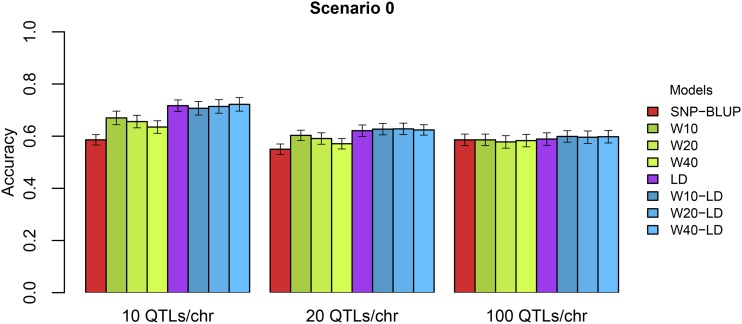
Accuracies obtained under different cases of genetic architecture of the trait for SNP-BLUP and the alternative models.

In order to investigate the performance of the alternative models for traits with different genetic architectures we simulated a trait controlled by an increasing number of QTL with each having a decreasing effect. As an overview, the accuracies of the different models in Sc0 with 20 and 100 QTL per chromosome are visualized in Figure 2 together with the results from 10 QTL per chromosome. The advantage of the alternative models over the SNP-BLUP model decreased with increasing number of QTL controlling the trait. When the number of QTL underlying the trait is 20 QTL per chromosome, the accuracies obtained were 9.6%, 7.5% and 3.8% better than the SNP-BLUP for the W10, W20 and W40 models, respectively. The continuous model resulted in a gain of 12.9% in accuracy while the combination models performed slightly better than all the alternative models yielding gains in accuracy of 14%, 14.2% and 13.5% for the W10-LD, W20-LD and W40-LD models, respectively. Finally, in the case of 100 QTL per chromosome, all models performed roughly the same as SNP-BLUP, yielding accuracies between 0.583 ± 0.012 (W40) and 0.599 ± 0.011 (W10-LD).

### Effect of variance of the QTL effects

**Figure 3:**
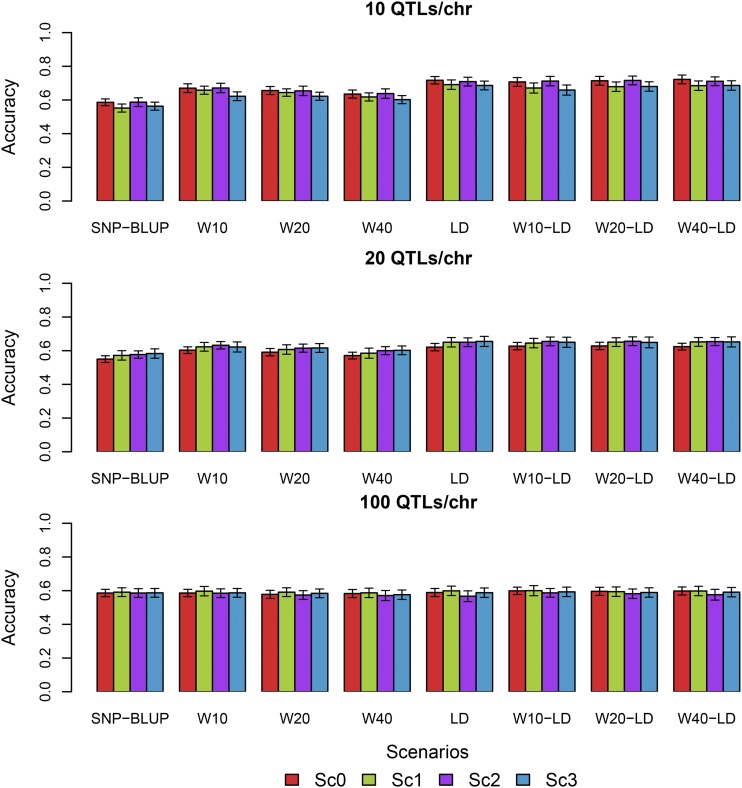
Accuracies obtained from SNP-BLUP model and alternative models under all simulated scenarios and genetic architectures.

The genetic architecture of a trait does not only depend on the number of QTL that affect the trait. For example, mutations can affect protein coding regions or regulatory regions and these mutations can have a bigger or smaller effect on the trait. Therefore we can assume that their effects come from a mixture of distributions with varying variance over the genome. For this purpose we simulated several scenarios where the QTL effects were drawn from a mixture of distributions (see Sc1 – Sc3 in Materials and Methods). We compared the performance of all models under all scenarios of QTL effect variances and all cases of number of QTL affecting the trait (Figure 3). In general the models performed similarly under Sc1, Sc2 and Sc3 as in Sc0. Small differences were observed in the case of 10 QTL per chromosome where all models performed slightly better in Sc0 and Sc2 (QTL effects from a low variance distribution on chromosome 1 and from high variance distribution on chromosome 2) compared with the results from Sc1 and Sc3. Nonetheless, this minimum difference disappeared quickly with increasing number of QTL per chromosomes. The external information included in the alternative models was related to the position of the QTL on the genome and/or the relationship of the SNPs with the QTL (LD), but no information about the distribution of the variance itself was included. Therefore, we fitted additional models that considered the way the QTL were simulated (see linear predictor 5: Additional models Material and Methods, and Supplementary file Table S1). For Sc1 and Sc2 we extended the linear predictor ($\alpha +\beta x_{j1}+\gamma x_{j2}$) to accommodate for two types of variances for the SNPs in windows that harbored a QTL assuming that we knew beforehand the distribution variance of the effect of that QTL and, as before, we tested 3 different window sizes (10, 20 and 40 SNPs per window). The results showed that these additional models performed similarly as the categorical models (W10, W20 and W40) under all cases of genetic architecture simulated. The only exception to these results was for the Sc2 with 100 QTL per chromosome where additional models showed a small increase in accuracy compared to all other models (Supplementary files, Figure S1). For the Sc3 we used the distance of the SNP from the edge of the chromosome as external information, either as a continuous variable or within windows. Similarly as before, the additional models that included information on the simulated distribution variance of the QTL did not perform better than the alternative models. The combined models (W10-Dis, W20-Dis and W40-Dis) performed the same as the categorical models while the continuous model (Dis) showed no benefit compared to the alternative models or the SNP-BLUP model under any simulation scenario of genetic architecture.

### Computation time

**Figure 4:**
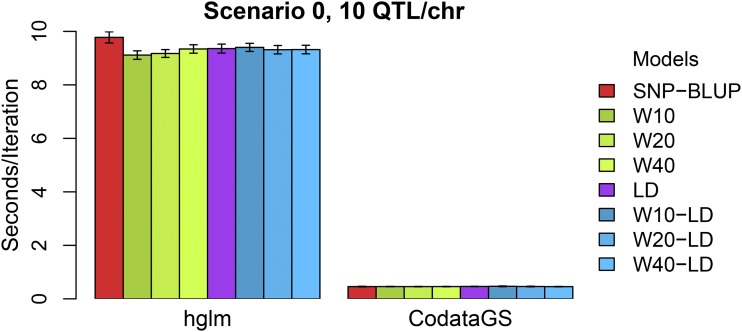
Time of execution (seconds per iteration) of SNP-BLUP and alternative models from hglm package and CodataGS package.

When the number of markers exceeds the number of individuals, the computational speed and memory requirements can be an important drawback for the use of such models. A solution to this problem is to fit individual effects (*i.e.*, EGBVs) in an equivalent model instead of SNP effects. In this study all evaluations were performed using the **hglm** R package that fits SNP effects. For a larger number of SNPs the computations would be unfeasible and an equivalent model which uses the external information on each SNP in the same way as in the **hglm** package was implemented in the R package **CodataGS** (https://cran.r-project.org/web/packages/CodataGS). The theory is explained in the Supplementary File S3. Fitting individual effects instead of SNP effects resulted in largely improved run time of all models. For a training population of 200 individuals with 2,000 SNP markers, fitting SNP effects (**hglm**) required on average 9.35 sec per iteration while fitting individual effects (**CodataGS**) required only 0.46 sec per iteration (Figure 4). The improved speed and memory requirements of the equivalent model can be considerably beneficial since the usual size of the training sets is much larger than the one used here (thousands of individuals with tens of thousands of SNPs). Nonetheless, the speed performance of the equivalent model depends heavily on the number of individuals and the relationship between time and number of individuals is not linear but rather exponential (Supplementary Figure S2).

### German Holstein population results

To demonstrate the model on real data, we used a German Holstein population consisting of 5024 bulls (Zhang *et al.* 2015). We chose to fit the model 1) **SNP-BLUP** and models 2) **W11** and **W41** with windows of sizes 11 and 41 SNPs centered around candidate QTL peaks. We obtained the candidate QTL peaks after performing a GWAS, correcting for genomic relationship using estimated residual and additive genetic variance from GBLUP. All SNPs from the GWAS with p-value less than 10¯^5^ were considered a candidate QTL. For milk yield (MY) we identified 6 candidate QTL peaks and for the fat percentage (Fat%) we identified 5 candidate QTL peaks, which were used as the center of the windows.

Table 3 shows the average accuracies obtained from the SNP-BLUP and W41 models for two traits (MY and Fat%) in the fivefold-cross-validation analysis. We present only the results from the W41 model as the model W11 yielded very similar accuracies as the W41 model. For both traits the W41 model yielded higher accuracies than the SNP-BLUP. The W41 model showed a higher advantage in predictive ability for the trait Fat% yielding an accuracy of 0.862 compared to the 0.811 obtained from the SNP-BLUP model. The results for the MY trait were similar but the predictive advantage of the W41 model was lower compared to the Fat% trait (accuracy of 0.785 from the W41 model over 0.771 from the SNP-BLUP model).

**Table 3:** MEAN ACCURACY (STANDARD ERROR) OF THE PREDICTED EGBVS IN A 5-FOLD CROSS VALIDATION ANALYSIS USING THE GERMAN HOLSTEIN DATA FOR TWO TRAITS

Models*^a^*	MY	Fat%
**SNP-BLUP**	0.771 ^(0.002)^	0.811 ^(0.004)^
**W41**	0.785 ^(0.002)^	0.862 ^(0.003)^

a W41= categorical model with window of 40 SNPs around the top SNP for the trait detected on a GWAS study. MY: Milk Yield, Fat%: Fat percentage.

## Discussion

The knowledge on the genetic architecture of different traits, and SNP-specific biological information, is increasing rapidly and several authors have proposed methods for genomic selection that can make use of this available biological information to improve selection accuracy (Zhang *et al.* 2010; Zhang *et al.* 2014; Su *et al.* 2014). In this line, this study proposes a general model using a link function approach within the hierarchical generalized linear model framework (Lee *et al.* 2006) to include biological external information into the model. Following Zhang *et al.* (2010), we used a base population of 100 individuals in our simulation study. This is a rather small population size and the results should therefore be extrapolated to larger effective population sizes with caution.

All the results in the current study use the same general model (described by equations 1 – 3) for predicting breeding values. The alternative models in Table 1, including SNP-BLUP, are fitted within this single framework and in the results the accuracies of the alternative models are compared. There are numerous Bayesian models not included within this framework that may be of interest to compare with. However, we use SNP-BLUP as a basic model to compare the results to and study the accuracies of models that make use of external information on the SNPs.

A very attractive feature of the method proposed in this study is that it provides a flexible way to model the SNP variances using a linear predictor (equation 3). Any type of existing knowledge on the SNP markers can be utilized and potentially increase the predictive ability of the model. In this study we investigated the performance of external information related to the position of the QTL on the genome and the relationship of the SNP markers with the QTL and we showed that the inclusion of such information can improve the predicting ability of genomic selection. From our results we identified two main factors that influence the performance of such models, the genetic architecture of the trait and the quality/accuracy of the external information.

In the models W10, W20 and W40, the causative effect is assumed to be within a window and does not assume that the exact position of the causative mutation is known. This model should be suitable for genomic prediction where external information from QTL studies is included. For the LD model and the combined models (W10-LD, W20-LD and W40-LD) it is assumed that the position of the causative SNP is known. Especially in plant breeding, there is a need to include major genes, whose positions are accurately known, in genomic prediction. For such cases the models including LD information combines marker assisted selection and genomic selection in a dynamic way.

We investigated models with three different window sizes that were suitable for our simulated data. For applications on real data the optimal number of markers to be included in each window, in terms of prediction accuracy, will depend on marker density and the genetic architecture. In our application on the dairy cattle data the optimal number of markers within a window was not assessed statistically, but since the marker map was much denser than in the simulated data we chose the model with the largest number of markers, *i.e.*, window size 40.

### Genetic architecture of the trait

The performance of several alternative models in our study was better compared to the SNP-BLUP method when the trait was controlled by a small number of QTL with medium-large effects. The advantage of these models was reduced with increasing number of QTL with smaller effects. However, the alternative models did not result in lower accuracies compared to the SNP-BLUP model. The reason is that as the estimated effect of the external information on the SNP variances approaches zero the model reduces to a SNP-BLUP model. Furthermore, as the number of QTL that control the trait increases, the external information on SNPs becomes more similar among the SNPs. For example, for the categorical models, a QTL is located within most or all defined windows and as a result all SNPs get the same weight in the model. Moreover, most or all SNPs are in LD with a QTL at similar levels. Consequently, the alternative models turn into a SNP-BLUP model. These results are in agreement with the findings of Zhang *et al.* (2010). In their simulation study they investigated the performance of a BLUP model with weighted G matrix and showed that for traits controlled by high number of QTL the traditional GBLUP and their method performed similarly. This effect has also been observed in studies on real data (Zhang *et al.* 2014). Analyzing three dairy cattle traits (Milk Yield (MY), Fat percentage (FP) and Somatic Cell Count (SCC)) these authors found that traits controlled by a few QTL with large effects (MY and FP) perform better under models with external information on the SNPs while the SCC trait, that is controlled by many QTL evenly distributed along the genome, performed better under the standard GBLUP model.

In our simulation study we created different genetic architectures for the trait with respect not only to the number of the QTL affecting the trait but also to the distribution of the QTL effects and their variances (see Material and Methods). Our results showed that this aspect did not affect the performance of the alternative models. Moreover, the additional models that included information on the variance distribution across the genome were not able to provide any benefit, contrary to methods that assume mixtures of distributions for the SNP markers like Bayesian methods (Erbe *et al.* 2012).

### External information

In this study we investigated the performance of models that include information on the location of the QTL on the genome (categorical models) and thereby tried to mimic the external information available on the QTL databases and the different window sizes resemble the degree of uncertainty of a QTL region. Our results indicate that this type of external information has the potential to improve the accuracy of genomic selection and that the degree of improvement is inversely related to the degree of uncertainty on the QTL region. The usefulness of the QTL database information has been demonstrated by Zhang *et al.* (2014). In their study these authors searched for reported QTL on the traits under consideration (Fat percentage, milk yield and somatic cell score for dairy cattle and several traits for rice) and after a quality control to avoid the possible false positive reports they included this information into a GBLUP model. For most of the examined traits an increase in accuracy was observed, especially for the traits that showed a characteristic genetic architecture. The discovery of new QTL or the causative mutations is expected to increase in the future with the use of whole genome sequence and the development of new methods for analysis and as a consequence the information available will become more accurate.

The external information that proved to be more valuable in this study was the LD estimates between the SNPs and the QTL. In the standard GBLUP method, markers in linkage equilibrium (LE) to the causative QTL tend to capture effects due to family relationship, whereas mainly markers in LD capture the QTL effects themselves (Habier *et al.* 2007, de los Campos *et al.* 2015). In the BayesB model (Meuwissen *et al.* 2001), the prior for the SNP variances is a mixture of two distributions that tends to group markers into two classes: those in LD and those in LE with the QTL. By modeling the two classes of markers better predictions for unrelated individuals can be obtained. In other studies, LD information has been incorporated in a model for the marker variances, which smooths the effects between markers in close LD (*e.g.*, the Bayesian antedependence model by Yang and Tempelman 2012, and the double hierarchical generalized linear model by Rönnegård and Lee 2010), and thereby captures the QTL effects rather than family information. These models give better predictions than GBLUP when individuals are unrelated and the total number of QTL is small. This is in line with our findings where the models including LD between markers and QTL resulted in improved prediction accuracies, especially when the number of simulated QTL was small. Finally, the results obtained from the combined models indicate that information on the real relationship between markers and QTL can compensate for the loss of information due to the uncertainty of the QTL report.

The prior of BayesB is rather general because it does not use any external information on the SNPs, whereas the model we propose gives more specific information about each SNP. Since the information on each SNP is more specific in our model its performance compared to GBPLUP and BayesB is expected to improve as the number of individuals in the training set decreases, in line with the results of Zhang *et al.* (2015, Supplementary Table 1) .

The model applied in Zhang *et al.* (2015) is BLUP|GA and was developed in Zhang *et al.* (2014). It includes external data on SNPs in the model and has similarities to our model since both methods fit trait-specific genomic relationship matrices. In the BLUP|GA method SNPs are divided into two groups by the user. In the first group there is a single genetic variance for all SNPs and in the second group SNP-specific variances are modeled as proportional to user-specific weights. Furthermore, the ratio between the variances for the two groups is also user-specified. This is indeed similar to our proposed method, but with some significant differences. The method that we propose uses a regression approach where covariates are specified by the user, whereas all model parameters are estimated. The covariates can include negative values in our method but the SNP variances will still be positive because the genetic variances are modeled using a logarithmic link function. By specifying covariates rather than weights for the SNP variances, hopefully, our proposed method will also be user friendly and the implementation in the CodataGS package (https://cran.r-project.org/web/packages/CodataGS) fits rather well with the regression framework in R.

### CONCLUSIONS

In this study we investigated the potential benefit of external information on improving the accuracy of genomic selection. In conclusion, using external information to model SNP-specific variances can provide gains in accuracy compared to the traditional SNP-BLUP. Nonetheless, the level of gain depends on the genetic architecture of the trait of interest and the quality of the external information on the SNP markers. The usefulness of these type of models is expected to increase with time as more accurate information on the SNPs becomes available. Finally, our analysis on real data indicated that the proposed method has potential but further studies are required to confirm the advantage of this approach.
